# Icariin Alleviates Wear Particle-Induced Periprosthetic Osteolysis *via* Down-Regulation of the Estrogen Receptor α-mediated NF-κB Signaling Pathway in Macrophages

**DOI:** 10.3389/fphar.2021.746391

**Published:** 2021-11-03

**Authors:** Fu Guangtao, Wen Zhenkang, Deng Zhantao, Li Mengyuan, Li Qingtian, Ma Yuanchen, Chen Yuanfeng, Zheng Qiujian

**Affiliations:** ^1^ Department of Orthopedics, Guangdong Provincial People’s Hospital, Guangdong Academy of Medical Sciences, Guangzhou, China; ^2^ Department of Orthopedics, Sun Yat-sen Memorial Hospital, Sun Yat-sen University, Guangzhou, China; ^3^ Research Department of Medical Science, Guangdong Provincial People’s Hospital, Guangdong Academy of Medical Sciences, Guangzhou, China

**Keywords:** icariin, wear particle, osteolysis, estrogen receptor α, macrophage

## Abstract

Periprosthetic osteolysis is one of the major long-term complications following total joint replacement. Its cause is widely accepted to be wear particle-induced activation of inflammatory macrophages. No effective strategy for the prevention and treatment of periprosthetic osteolysis is yet available. Recently, considerable evidence has shown that icariin effectively protects against estrogen deficiency-related bone loss and bone deterioration. However, the molecular mechanism underlying the inhibitory effect of icariin on wear particle-induced periprosthetic osteolysis is not yet clear. In this study, nanoscale CoCrMo wear particles were obtained by high-vacuum three-electrode direct current from the femoral head implant of a patient diagnosed with aseptic loosening. The effects of icariin on wear particle-induced expression of proinflammatory factors, NF-κB signaling modulation, osteolysis, and estrogen receptor α (ERα) activation were evaluated *in vitro* and *in vivo* using bone marrow-derived macrophages and C57/BL6J mice, respectively. A possible link between ERα and the protective effect of icariin was further studied using an ERα antagonist and the ERα-siRNA interference. Chemical composition analysis showed that Cr and Co were the major metallic elements of the nanoscale particles, with a mean size of 150.2 ± 37.4 nm for the CoCrMo particles. Following icariin treatment, significant decreases were observed in CoCrMo wear particle-induced TNF-α and IL-6 mRNA expression in BMDMs, and osteolysis in mice calvaria. Marked decreases in the protein expression level of p-IKKβ, p-p65 and p-IκBα were also observed, together with significant decreases in the nuclear import of P65 and macrophage M1 polarization. RNA sequencing revealed that ERα was closely associated with TNF-α and IL-6 in wear particle-stimulated macrophages. Furthermore, marked increases in phospho-ERα Ser118 and phospho-ERα Ser167 protein expression and the nuclear import of ERα were also found in the icariin group. The protective effects of icariin on CoCrMo particle-induced mouse calvarial osteolysis and on the inflammation response in BMDMs were reversed by ERα antagonist and by ERα-siRNA interference. In conclusion, icariin attenuates wear particle-induced inflammation and osteolysis *via* down-regulation of the ERα-mediated NF-κB signaling pathway in macrophages. The potential application of icariin as a non-hormonal therapy for wear particle-induced periprosthetic osteolysis is worthy of further investigation.

## Introduction

Periprosthetic osteolysis is one of the major long-term complications following total joint replacement and is associated with aseptic implant failure and severely impaired joint function (Goodman et al., 2020). Demographic data show that 16.2% of patients who underwent primary total hip replacement were suffering from periprosthetic osteolysis-related aseptic implant loosening at the 16th postoperative year (Taylor et al., 2018). Since there are over 5 million total joint replacements annually (Pincus et al., 2020), the public health and economic burden of periprosthetic osteolysis has emerged as a major issue.

Wear particles released from the surface of implants are the main cause of periprosthetic osteolysis. After being phagocytized by macrophages in the surrounding synovial tissue, wear particles induce marked activation of the inflammatory response and subsequent osteoclastogenesis (Goodman and Gallo, 2019). The pattern recognition receptor on the macrophage membrane and the downstream protein kinase cascade signaling pathways (MAPK, PI3k/Akt/NF-κB, etc.) are heavily involved in wear particle-induced chronic inflammatory and periprosthetic osteolysis (Goodman et al., 2020). Consequently, these have been proposed as potential targets for novel medical therapies (Hu et al., 2020). There is currently no effective medical therapy for the prevention and treatment of periprosthetic osteolysis.

Estrogen deficiency is a common comorbidity in patients undergoing total joint replacement, being present in nearly 60% of cases (James et al., 2014). Interestingly, a link has been described between estrogen deficiency and wear particle-induced osteolysis. We previously reported significantly higher postoperative periprosthetic bone loss and wear particle-induced mice calvarial osteolysis in conditions of estrogen deficiency, indicating that estrogen has a protective effect against wear particle-induced periprosthetic osteolysis (Fu et al., 2018; Fu et al., 2019).

Icariin (ICA) is a major component of the Chinese herb *Herba Epimedii*, which is commonly prescribed for the treatment of estrogen deficiency-induced osteoporosis (Ho et al., 2018). ICA is a bone bioactive flavonoid and phytoestrogen that exerts estrogen-like bone protective effects without inducing estrogenic effects in the uterus and breast (Zhou et al., 2020). Clinical and animal studies have shown that ICA protects against estrogen deficiency-related bone loss and bone deterioration (Zhang et al., 2007; Zhou et al., 2021). Furthermore, the *in vitro* osteogenic and anti-osteoclastic effects of ICA on bone marrow stromal cells (Xu et al., 2020; Zhou et al., 2021), osteoblasts (Ho et al., 2018) and macrophages (RAW264.7 cells) (Kim et al., 2018) have also been extensively documented. Recently, an animal study reported that ICA significantly suppressed wear particle-induced osteoclastogenesis, local inflammation response, and osteolysis in mice calvarias by inhibiting NF-κB signaling pathway (Shao et al., 2015). Another *in vitro* study also found that ICA inhibited wear particles-induced osteoclastogenesis *via* the suppression of NF-κB signaling in RAW264.7 cells (Cui et al., 2014). However, the underlying mechanism and upstream regulators of ICA induced macrophage NF-κB signaling inhibition in the circumstance of wear particle stimulation remains unclear. The aim of the present study was therefore to investigate the molecular mechanism and related regulatory pathways that underlie the inhibitory effect of ICA on wear particle-induced osteolysis and inflammation response in macrophages. This was achieved using both *in vivo* and *in vitro* experiments.

## Materials and Methods

### Clinical Specimen Collection

Synovial membranes from the involved hip were obtained from four patients who underwent total hip revision in our institute from Jan 1st, 2019 to Dec 31st, 2019 due to aseptic loosening of their implant. Samples of synovial membranes were also obtained from four patients who underwent primary total hip replacement or hemiarthroplasties because of hip fracture. Demographic data for these eight patients are shown in Supplementary Table S1. This study was approved by the institutional review board of our hospital. Signed informed consent for participation and publication were obtained from all patients and their relatives.

### Particle Preparation

Wear particles were made from the femoral head implant (CoCrMo alloy, Modular prosthesis head, Link) of patient No. 2 (details shown in Supplementary Table S1) who was diagnosed with aseptic loosening. All experiments were performed according to Chinese legal requirements. The removed femoral head was sterilized and subsequently transformed into nanoscale wear particles using a fabricated high-vacuum three-electrode direct current previously described by our group (Deng et al., 2021). The appearance of the wear particles was assessed by field emission scanning electron microscopy (Merlin, Carl Zeiss AG, Germany) and by transmission electron microscopy (JEM-2100, JEOL, Japan). Size distribution analysis was performed with a Nano Sizer and zeta-potential tester (Zetasizer Nano ZS, Malvern, United Kingdom). The chemical composition of wear particles was analyzed using tungsten filament scanning electron microscopy (Q25, FEI, OR, United States). All wear particles were washed with 75% ethanol and sterilized with ethylene oxide. The absence of endotoxin was confirmed using a commercial limulus assay kit (QCl-1000; Bio Whittaker, Walkersville, MD, United States). CoCrMo particles were then suspended in PBS and adjusted to a concentration of 8 × 10^–3^ g/ml for *in vitro* experiments.

### Bone Marrow-Derived Macrophage Culture

Bone marrow cells were collected from the femur and tibia of 12-week-old C57BL/6J female mice. BMDMs were obtained after co-culture of bone marrow cells in Dulbecco’s modified Eagle’s medium containing 10% fetal bovine serum, 10 ng/ml recombinant M-CSF, penicillin, and streptomycin for 7 days. All BMDMs were cultured at 37°C in 5% CO_2_, followed by seeding for 24 h in high-glucose Dulbecco’s modified Eagle’s medium containing 10% fetal bovine serum, penicillin, and streptomycin. Cell counters (Countstar BioTech) were used to show at least 98% cell viability in all trials. The identification of BMDMs was performed by flow cytometry analysis with the marker being F4/80 + CD11b+.

### Real-Time PCR and ELISA

ICA was administrated simultaneously with wear particles in both PCR and ELISA testing. At specific time points after stimulation, RNA was extracted using an RNA extraction kit according to the manufacturer’s instructions (9109, TaKaRa Biotechnology). PrimeScript RT Master Mix (RR036D, TaKaRa Biotechnology) was used to reverse-transcribe RNA to cDNA. Real-time PCR was performed using UNICONTM qPCR SYBR Green Master Mix (11198ES08, Yeasen) and a LightCycler 96 real-time PCR system (Roche Molecular Systems, Inc) to measure the expression of TNF-α and IL-6. GAPDH was used as the housekeeping gene. All reactions were run in triplicate. Primers for the target genes are listed in Supplementary Table S2.

For ELISA measurement, the cell supernatants were harvested and centrifuged to remove cellular debris. TNF-α and IL-6 levels were subsequently assessed using an instant enzyme-linked immunosorbent assay (ELISA) kit (Neobioscience Technology Co., Ltd) according to the manufacturer’s instructions.

### Western Blot

Following stimulation by wear particles for 1 h and other interventions, BMDMs were lysed using a RIPA buffer (9806S, Cell Signaling Technology) containing PMSF (P0100-1, Solarbio) and a phosphatase inhibitor cocktail (CW2383, CW Biotech). Proteins were separated by 10% sodium dodecyl sulfate-polyacrylamide gel electrophoresis and then transferred to polyvinylidene fluoride membranes. The membranes were cut into small pieces according to different protein molecular masses and then probed with the relevant antibodies overnight at 4°C. Details of the antibodies are listed in Supplementary Table S3. A chemiluminescence detection system (Syngene, Cambridge, United Kingdom) was used to visualize the protein bands and further analysis was performed using Gene Tools (Syngene, Cambridge, United Kingdom).

### Immunofluorescence Staining

One hour after being stimulated with wear particles, BMDMs were incubated with p65 (1:400, rabbit, 8242, Cell Signaling Technology) or ERα (1:200, rabbit, ab32063, abcam) antibodies overnight at 4°C. Subsequently, BMDMs were washed three times with PBS followed by incubation with secondary IF antibody (goat anti-rabbit, A32740, Thermo Fisher Scientific) at 20°C for 1 h. Thereafter, BMDMs were treated with an anti-fade, fluorescence mounting medium with DAPI (HNFD-02, HelixGen Co., Ltd). A confocal microscope (LSM 710, Carl Zeiss) was used to visualize IF.

### Flow Cytometry

BMDMs were stimulated by wear particles for 24 h and then treated with TrypLE Express Enzyme (12604021, Thermo Fisher Scientific) to detach cells from the plates. They were then washed with Perm/Wash buffer, suspended in PBS and analyzed using flow cytometry (BD FACSVerse, BD Biosciences). After centrifugation at 1,000 rpm for 5 min, BMDMs were sequentially incubated with APC-conjugated CD206 antibody (0.25ug/test, 17-2061-82, Thermo Fisher Scientific) and PE-cyanine7-conjugated iNOS antibody (0.06ug/test, 25-5920-82, Thermo Fisher Scientific). The isotype controls used were APC-conjugated rat IgG2b, κ (17-4031-82, Thermo Fisher Scientific) and PE-cyanine7-conjugated rat IgG2a, κ (25-4321-82, Thermo Fisher Scientific).

### SiRNA Synthesis and Lentivirus Transfection

To generate ERα knockdown macrophages, siRNA that targets Esr1 (GeneID: 13982) was constructed into the plasmid and recombinant lentiviruses aimed at Esr1 were obtained from Cyagen. The specific siRNA sequences are listed in Table 1. For transient transfections, 5×10^5^ BMDMs were seeded for 24 h and nurtured in Opti-MEMI Reduced Serum Medium (31985070, Thermo Fisher Scientific) for 12 h. Subsequently, 200 pmol siRNA together with 7.5 μL Lipofectamine RNAiMAX Transfection Reagent (13778075, Thermo Fisher Scientific) were added for a 24 h transfection period and the ERα knockdown efficiency then evaluated by qPCR assay. For the production of stable ERα knockdown macrophages, BMDMs were cultivated with 1 × 10^7^ transducing unit per mL lentiviruses for 24h and 3ug/mL puromycin was used to screen the transfected macrophages.

**TABLE 1:** Sequences of ERα-shRNAs.

Sequence	Gene sequence (5 to 3′)
shRNA-ERα#1	CCC​ATG​ATC​TAT​TCT​GAA​TAT​CTC​GAG​ATA​TTC​AGA​ATA​GAT​CAT​GGG
shRNA-ERα#1	CCG​CCT​TCT​ACA​GGT​CTA​ATT​CTC​GAG​AAT​TAG​ACC​TGT​AGA​AGG​CGG
shRNA-ERα#1	GCT​TTC​TTT​AAG​AGA​AGC​ATT​CTC​GAG​AAT​GCT​TCT​CTT​AAA​GAA​AGC
Negative control	UUC​UCC​GAA​CGU​GUC​ACG​UTT ACG​UGA​CAC​GUU​CGG​AGA​ATT
GAPDH	CAC​UCA​AGA​UUG​UCA​GCA​ATT UUG​CUG​ACA​AUC​UUG​AGU​GAG

### Protein-Protein Interaction Network Construction and Identification of Hub Genes

RNA sequencing was performed after the RAW264.7 macrophages had been stimulated by wear particles for 4 h. PPI network construction and identification of hub genes has been described previously (Qiu et al., 2020). Briefly, the STRING database and Cytoscape software were used to construct a PPI network of differentially expressed genes. The topology property of the network was analyzed using the MCODE application of Cytoscape software. The functional clustering of differentially expressed genes was performed using the Metascape online tool (https://metascape.org).

### Wear Particle-Induced Mouse Calvaria Osteolysis Model

Twenty-five C57/BL6J female mice aged 10 weeks were purchased from the Laboratory Animal Research Center of the South China University of Technology. All experiments were approved by the Institutional Animal Care and Use Committee, Guangdong Province People’s Hospital. Guidelines for the care and use of laboratory animals were strictly followed. Each mouse weighed 20–25 g at the beginning of the study, and all had unlimited access to food and water.

The wear particle-induced mouse calvaria osteolysis model was established when mice reached the age of 12 weeks. The mice were divided into 5 groups: control (particle-free), wear particle (Pa), wear particle + icariin (Pa + ICA), wear particle + icariin + ERα antagonist methyl-piperidino-pyrazole (MPP) (Pa + ICA + MPP), and wear particle + icariin + ERα-siRNA (Pa + ICA + siRNA) group. There were five C57BL/6J mice in each group. As we previously described (Fu et al., 2018), the center of the calvaria was exposed and the periosteum removed. In the Pa, Pa + ICA, and Pa + ICA + MPP groups, 50 ul of PBS containing 0.3 mg wear particles was spread onto the center of the calvaria. In the Pa + ICA + siRNA group, 50 ul PBS containing 0.3 mg wear particles, as well as 25 ul shRNA-ERα lentivirus (titer: 1×10^8^ TU/mL) were locally spread onto the center of the calvaria before suturing according to our established method (Zhang et al., 2018). In the Pa + ICA, Pa + ICA + MPP, and Pa + ICA + siRNA groups, ICA (2.8 mg/50 μL, B21576, YuanYeBio, China) and MPP (5.6 μg/50 μL, M7068, Sigma) were continuously and subcutaneously delivered by minipumps (micro-osmotic pump, model 1,004, 0.11 μL/h*28 days, Alzet, United States). The dosages of ICA and MPP used in this animal model were obtained from previous studies (Davis et al., 2008; Shao et al., 2015). Minipumps were subcutaneously implanted at a site slightly posterior to the scapulae. No mice died and no wound complications were observed during the experiments. All mice were sacrificed in a CO_2_ chamber at day 14 after surgery.

### Micro-CT Imaging

After removing all the soft tissue, the calvarias were analyzed by micro-CT and associated analysis software (SkyScan 1176; SkyScan, Kontich, Belgium). The scanning protocol was set at an isometric resolution of 18 μm and radiographs were acquired at 45 kV and 500 uA through a 0.2 mm-thick aluminum filter with an exposure time of 240 ms. 3D image reconstructions were obtained using manufacturer’s software (NRecon, SkyScan, Kontich, Belgium). The region of interest (ROI) was set as previously described (Fu et al., 2018). BV/TV, BMD and the total porosity of each sample were obtained using CTAn software (SkyScan, Kontich, Belgium).

### Histological and Immunohistochemical Analysis

Cross-sections (4 µm) of the calvarias were cut and stained with hematoxylin and eosin (H&E) and with a commercial tartrate-resistant acid phosphatase (TRAP) staining kit (Jiancheng Bio Ins, Nanjing, China). Histological sections were analyzed using a standard, high-quality light microscope (DM 2000, Leica, Germany) at a magnification of 10X with the midline suture in its center. Histomorphometric analysis was performed on the most central section and on four adjacent sections using image analysis software (Image Pro-Plus 6.0, Media Cybernetics, Bethesda, MD). The definition of ROI, as well as measurements of the eroded bone area (mm^2^) and number of osteoclasts was performed as per earlier studies (Sawyer et al., 2003; von Knoch et al., 2004). Briefly, the eroded bone area (mm^2^) was determined by tracing the area of soft tissue between the parietal bones, including resorption pits on the superior surface of the calvaria. The presence of dark-purple-stained granules in the TRAP staining section, which is located on the bone perimeter within a resorption lacuna, was automatically recognized as osteoclasts by the image analysis software after manual setting of the color parameters.

The protocol for histological detection of TNF-α, IL-6, and ERα in human synovial membranes and mice calvarias was as follows. All sections first underwent endogenous peroxidase blocking and antigen retrieval. After being incubated with goat anti-mouse IgG, sections were then incubated overnight at 4°C with primary antibodies for TNF-α (ab183218, abcam), IL-6 (ab233706, abcam), and ERα (ab32063, abcam). Following incubation with the appropriate secondary antibodies, coloration of the sections was achieved with 3,3-diaminobenzidin. Rinsed sections were counterstained with hematoxylin. Three views of each section with the magnification of 100x were evaluated and the Bresalier’s semiquantitative scoring system was used to quantify the staining intensity (Bresalier et al., 1997). Briefly, the staining intensity (0 for no stain; 1 for weak stain; 2 for moderate stain; 3 for strong stain) and the percentage of cells with specific staining intensity (P0, P1, P2, P3) of each section were calculated independently by two pathologists. Subsequently, the immunoreactivity score for each section was calculated as follows: ∑(0×P0+1×P1+2×P2+3×P3).

### Statistics

All the results of *in vitro* studies were obtained from at least three independent experiments, with data expressed as mean ± SD. The values were first assessed using the Kolmogorov-Smirnov test to verify data normality. The two-sided Student’s t-test was used to compare two groups. One-way analysis of variance was used for three or more groups, and Tukey test was used for post hoc comparisons. Statistical analyses were performed using SPSS 20.0 software, with *p* < 0.05 considered to be statistically significant.

## Results

### Physical Characteristics of the Wear Particles

**FIGURE 1:**
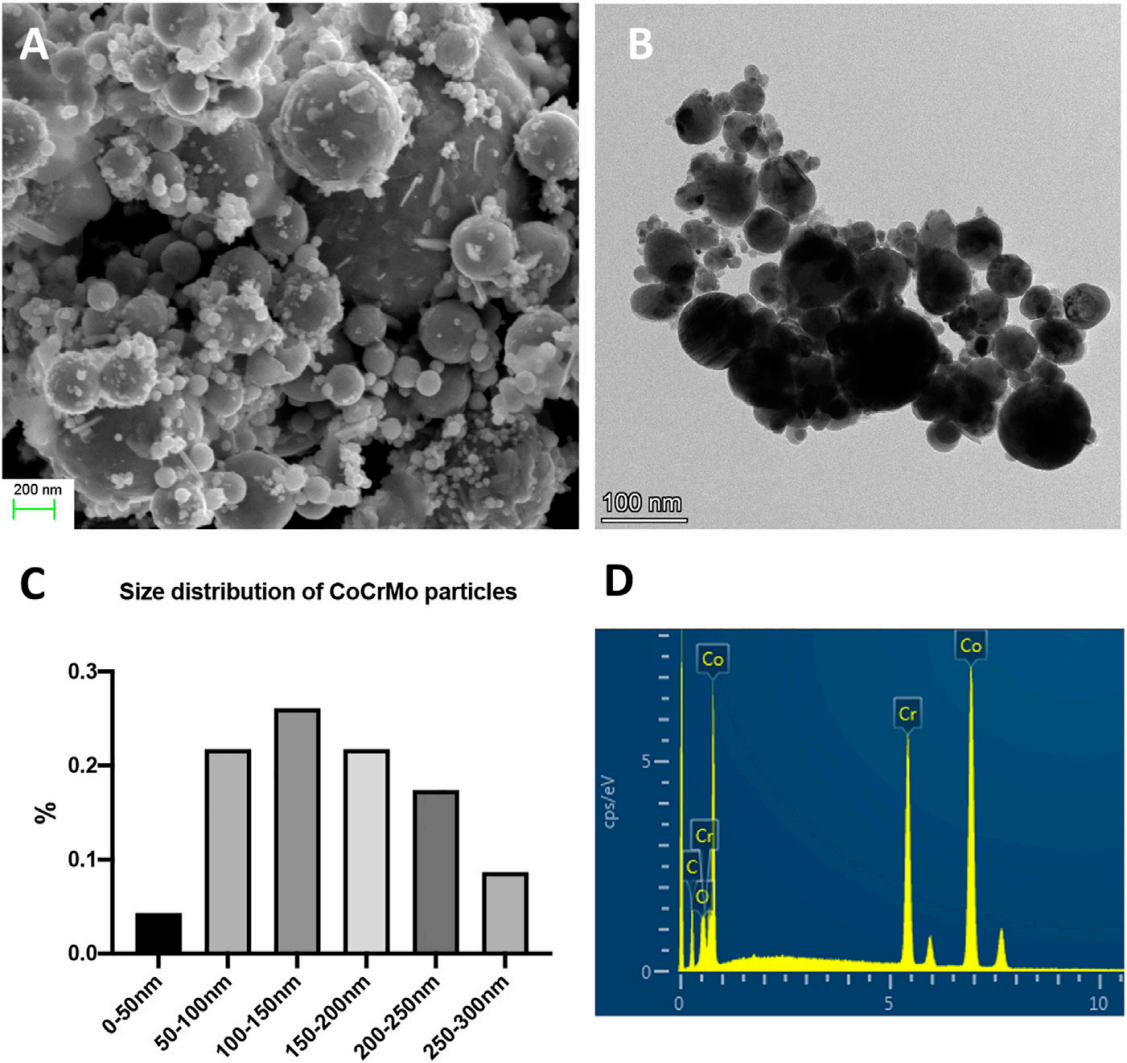
The physical characteristics of CoCrMo nanoparticles derived from the femoral head implant of a patient who was diagnosed with aseptic loosening. **(A)** Scanning electron microscopy image of CoCrMo nanoparticles. **(B)** Transmission electron microscopy image of CoCrMo nanoparticles. **(C)** Size distribution of CoCrMo nanoparticles. **(D)** Chemical composition of CoCrMo nanoparticles.

Scanning electron microscopy and transmission electron microscopy images of the nanoscale wear particles are shown in Figures 1A,B. The mean size of the particles was 150.2 ± 37.4 nm and their size distribution is shown in Figure 1C. The chemical composition of the wear particles was shown in Figure 1D and the normalized mass of each element was available in Table 2. Consistent with the known composition of the CoCrMo alloy femoral head (Link), the leading metallic elements of the nanoscale wear particles were Cr and Co.

**TABLE 2:** Quantified measurements of the chemical composition of CoCrMo nanoparticles.

Element		Cr	Co	C	O	Ti	Ni
—	Sample 1	22.95	59.07	16.68	1.29	0	0
Normalized mass (%)	Sample 2	13.91	60.86	12.59	4.04	0.34	8.27
—	Sample 3	22.15	69.95	7.14	0.76	0	0
Mean		19.67	63.29	12.14	2.03	0.11	2.76
SD		4.09	4.76	3.91	1.44	0.16	3.90

### CoCrMo Wear Particles Induce TNF-α and IL-6 Expression *via* NF-κB Signaling Activation

**FIGURE 2:**
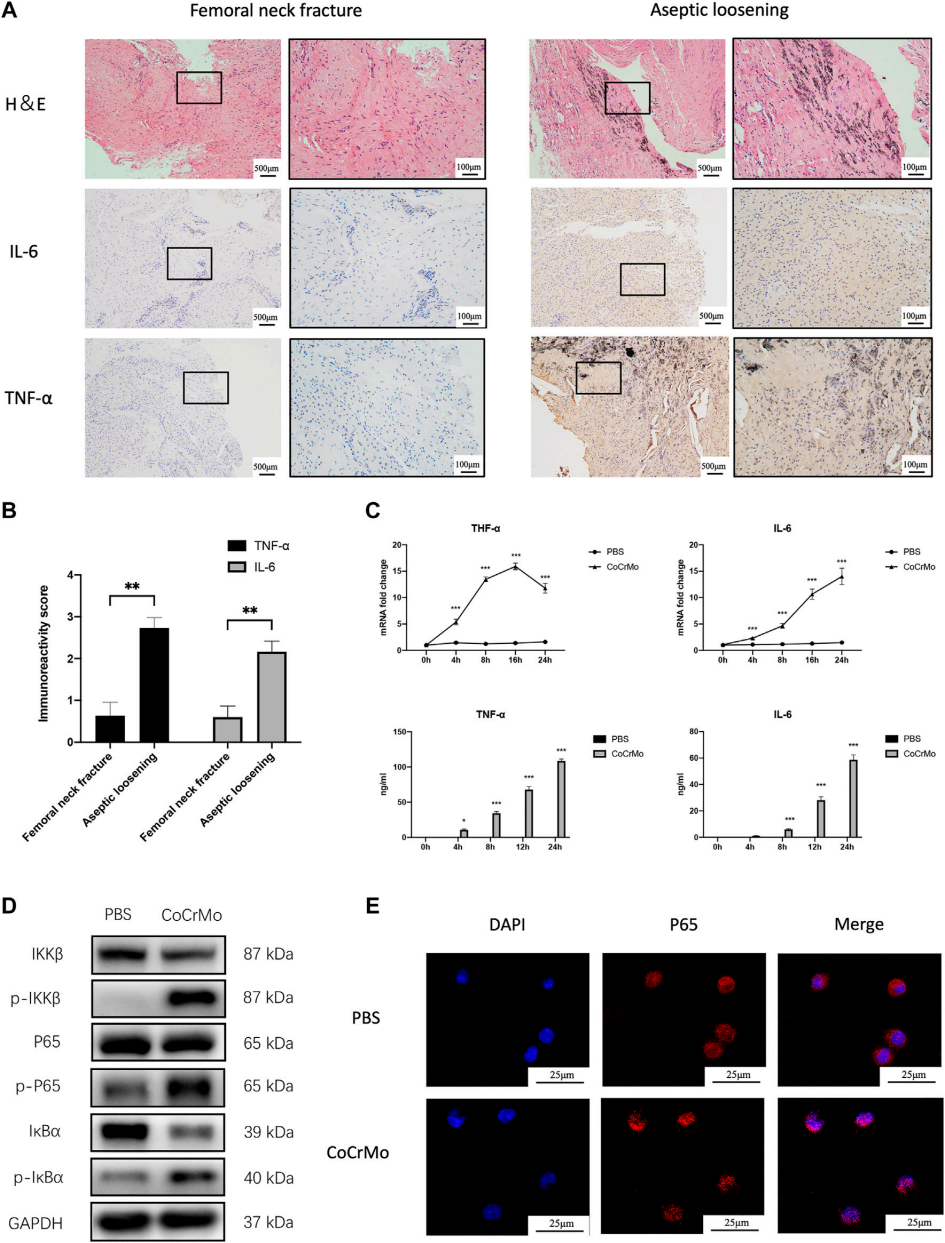
CoCrMo wear particles induced TNF-α and IL-6 expression *via* NF-κB signaling activation. **(A)** H&E staining and immunohistochemical staining of TNF-α and IL-6 in synovial membranes of four patients who were diagnosed with aseptic implant loosening and another four patients who were diagnosed with femoral neck fracture. **(B)** Semiquantitative assessments of the staining intensity using Bresalier’s semiquantitative scoring system for the aseptic implant loosening group and femoral neck fracture group. **(C)** mRNA expressions and secretions of TNF-α and IL-6 by BMDMs were significantly higher than the PBS group after being stimulated by CoCrMo wear particles. **(D)** Increased protein concentrations of p-IKKβ, p-p65 and p-IκBα in BMDMs following CoCrMo particle stimulation. **(E)** p65 protein (red) translocates from the cytoplasm into the nucleus (blue) of BMDMs after being stimulated by CoCrMo wear particles. * indicates *p* < 0.05, ** indicates *p* < 0.01, and *** indicates *p* < 0.001 when compared with the control group at the same time point.

Marked wear particle infiltration was found in H&E-stained sections of the synovial membranes obtained from the involved hip of patients diagnosed with aseptic loosening (Figure 2A). Immunohistochemical staining of the synovial membranes revealed significantly higher staining intensities for TNF-α (+2.10 ± 0.37, *p* < 0.01) and IL-6 (+1.57 ± 0.29, *p* < 0.01) in the aseptic loosening group, as calculated by Bresalier’s semiquantitative scoring system (Figures 2A,B).

Results of flow cytometry for the identification of BMDMs were provided in Supplementary Figure S1. TNF-α and IL-6 mRNA levels increased in a time-dependent manner following stimulation of BMDMs by CoCrMo wear particles (Figure 2C). Similarly, ELISA revealed that TNF-α and IL-6 secretion by BMDMs increased significantly and gradually after 4, 8, 12 and 24 h stimulation by wear particles. To further investigate the molecular mechanism underlying wear particle-induced activation of inflammation, WB was used to evaluate protein expression for NF-κB signaling pathway components. Marked increases in the expression of p-IKKβ, p-p65 and p-IκBα were observed, but no obvious changes in the total protein expression of IKKβ, p65 and IκBα (Figure 2D). Confocal microscopy also revealed translocation of p65 protein (red) from the cytoplasm into the nucleus (blue) following CoCrMo particle stimulation of BMDMs (Figure 2E).

### ICA Inhibits CoCrMo Wear Particle-Induced Activation of NF-κB Signaling and Proinflammatory Factor Expression in BMDMs

**FIGURE 3:**
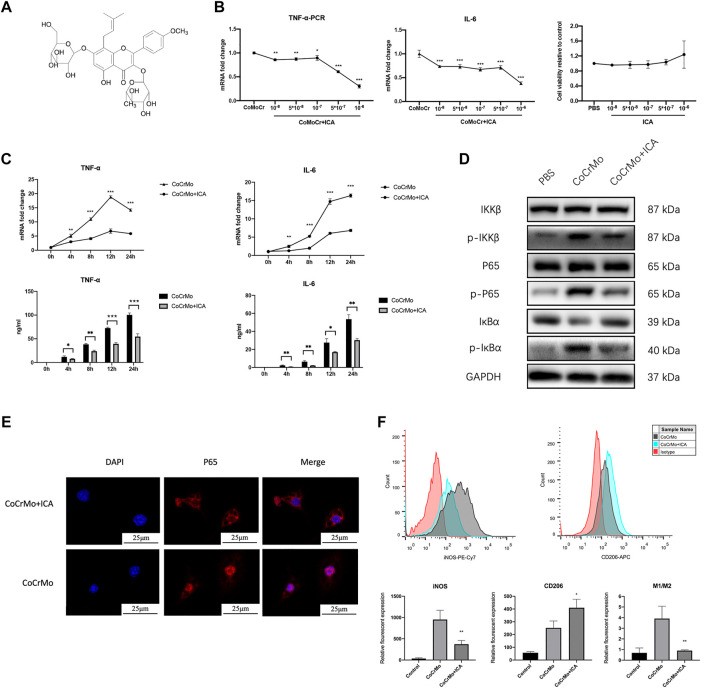
ICA suppressed CoCrMo wear particle-induced activation of NF-κB signaling and the expression of proinflammatory factors in BMDMs. **(A)** The chemical structure of ICA. **(B)** The inhibitory effect of ICA on CoCrMo wear particle-induced TNF-α and IL-6 mRNA expression in BMDMs was dose-dependent (10^−8^ to 10^−6^ M). No significant changes in cell viability were observed when the ICA concentration was varied from 10^−8^ to 10^−6^ M. **(C)** The mRNA expression and secretion of TNF-α and IL-6 in BMDMs decreased significantly in the ICA group. **(D)** Marked decreases in p-IKKβ, p-p65 and p-IκBα protein expression were observed after ICA treatment. **(E)** CoCrMo-induced translocation of p65 (red) from the cytoplasm into the nucleus (blue) was reduced in the CoCrMo + ICA group. **(F)** A decreased mean fluorescent expression of iNOS and an increased mean fluorescent expression of CD206 were found after ICA treatment. * indicates *p* < 0.05, ** indicates *p* < 0.01, and *** indicates *p* < 0.001 when compared with the control group at the same time point.

Figure 3A shows the chemical structure of ICA. The inhibitory effect of ICA on CoCrMo wear particle-induced expression of TNF-α and IL-6 mRNA in BMDMs occurred in a dose-dependent manner (10^−8^ to 10^−6^ M), as shown in Figure 3B. The maximum inhibitory effect of ICA was found at a concentration of 10^−6^ M. CCK-8 assay was also performed to determine the influence of ICA concentration on the proliferation and survival of BMDMs. No significant changes in cell viability were observed at ICA concentrations ranging from 10^−8^ to 10^−6^ M (Figure 3B).

ICA was therefore used at a concentration of 10^−6^ M in the subsequent *in vitro* experiments. Compared to the CoCrMo group, the CoCrMo + ICA group showed significantly lower TNF-α and IL-6 mRNA levels and protein secretion at 4, 8, 12 and 24 h after CoCrMo particle stimulation (Figure 3C). These results suggest that the inhibitory effect of ICA on the CoCrMo wear particle-induced macrophage inflammatory response was related to the downregulation of NF-κB signaling pathways. Marked decreases in p-IKKβ, p-p65 and p-IκBα protein expression were observed after ICA treatment (Figure 3D). IF staining showed that CoCrMo-induced translocation of p65 (red) from the cytoplasm into the nucleus (blue) was consistently inhibited in the CoCrMo + ICA group (Figure 3E). Activation of the NF-κB pathway is thought to be responsible for M1 polarization of macrophages, which is pivotal for the wear particle-induced inflammatory response. Therefore, we also evaluated macrophage polarization in the CoCrMo and CoCrMo + ICA groups using flow cytometry. Following ICA treatment, a significantly lower mean fluorescent expression (-58.7 ± 12.2%, *p* < 0.01) of iNOS and a significantly higher mean fluorescent expression (70.8 ± 50.6%, *p* < 0.05) of CD206 were observed (Figure 3F).

### The Inhibitory Effect of ICA on the CoCrMo Particle-Induced BMDM Inflammatory Response Was Mediated by ERα

**FIGURE 4:**
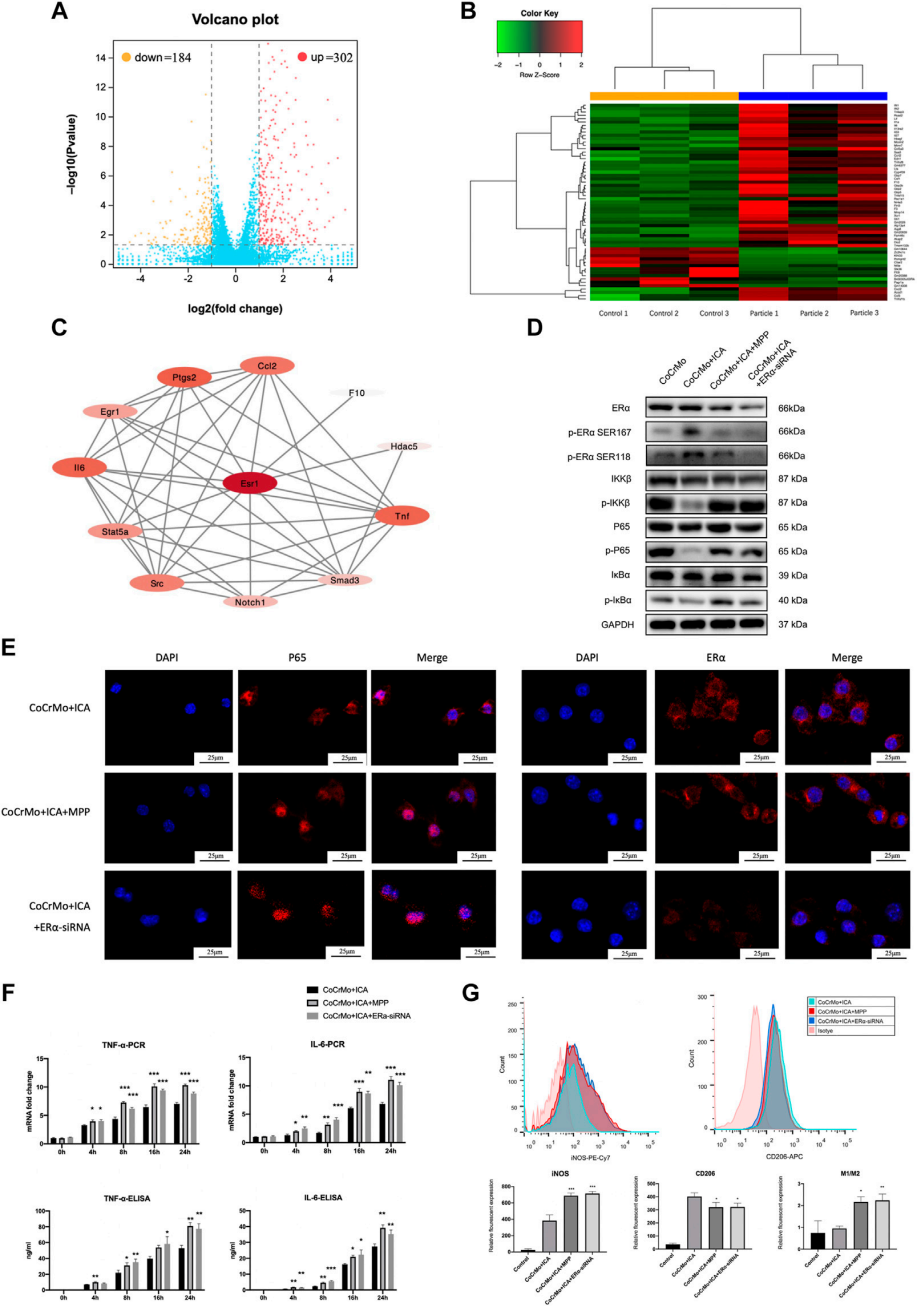
The inhibitory effect of ICA on the CoCrMo particle-induced BMDM inflammatory response was mediated by ERα. **(A)** Volcano plot shows a total of 302 upregulated mRNAs and 184 downregulated mRNAs in macrophages after stimulation by CoCrMo particles. **(B)** The heat map. **(C)** The PPI network from the STRING database showed that ERα has a close relationship with TNF-α and IL-6. **(D)** The inhibitory effect of ICA on p-IKKβ, p-p65 and p-IκBα protein expression was blocked by ERα antagonist and by ERα-siRNA interference. **(E)** Confocal microscopy showing translocation of ERα from the cytoplasm into the nucleus after ICA treatment, whereas P65 translocation toward the nucleus was suppressed. **(F)** No significant differences in the mRNA transcription and protein secretion of TNF-α and IL-6 by BMDMs were found between the CoCrMo, CoCrMo + ICA + MPP, and CoCrMo + ICA + ERα-siRNA groups. **(G)** The effect of ICA on CoCrMo particle-induced macrophage M1 and M2 polarization was reversed by ERα antagonist and by ERα-siRNA interference. * indicates *p* < 0.05, ** indicates *p* < 0.01, and *** indicates *p* < 0.001.

RNA sequencing and PPI network construction was performed to further investigate the underlying mechanism of wear particle-induced osteolysis and to identify potential hub genes. The volcano plot and heat map for this analysis are shown in Figures 4A,B, respectively. A total of 302 upregulated mRNAs and 184 downregulated mRNAs were profiled (log 2 FC > 1, FDR <0.05). The PPI network from the STRING database showed that ERα has a close relationship with TNF-α and IL-6 (Figure 4C).

As shown in Figure 4D, ICA induced a marked increase in the protein expression of both phospho-ERα Ser118 and phospho-ERα Ser167 while inhibiting the wear particle-related macrophage inflammation response. Confocal microscopy also showed translocation of ERα from the cytoplasm into the nucleus following ICA treatment, whereas P65 translocation towards the nucleus was suppressed (Figure 4E).

Furthermore, both ERα antagonist (MPP) and ERα-siRNA interference reversed the suppressive effect of ICA on the CoCrMo particle-induced mRNA transcription and protein secretion of TNF-α and IL-6 in BMDMs (Figure 4F). Similar changes were also noted for activation of the NF-κB signaling pathway. No significant differences in p-IKKβ, p-p65 or p-IκBα protein expression were found between the CoCrMo, CoCrMo + ICA + MPP and CoCrMo + ICA + ERα-siRNA groups (Figure 4D). Consistent with this, the inhibitory effect of ICA on CoCrMo particle-induced translocation of P65 from the cytoplasm into the nucleus was reversed by both ERα antagonist and ERα-siRNA interference (Figure 4E). Similar trends were also observed regarding the influence of these two interventions on macrophage polarization (Figure 4G).

### The Protective Effect of ICA on CoCrMo Particle-Induced Mouse Calvarial Osteolysis and Local Inflammatory Response Was Regulated by ERα

**FIGURE 5:**
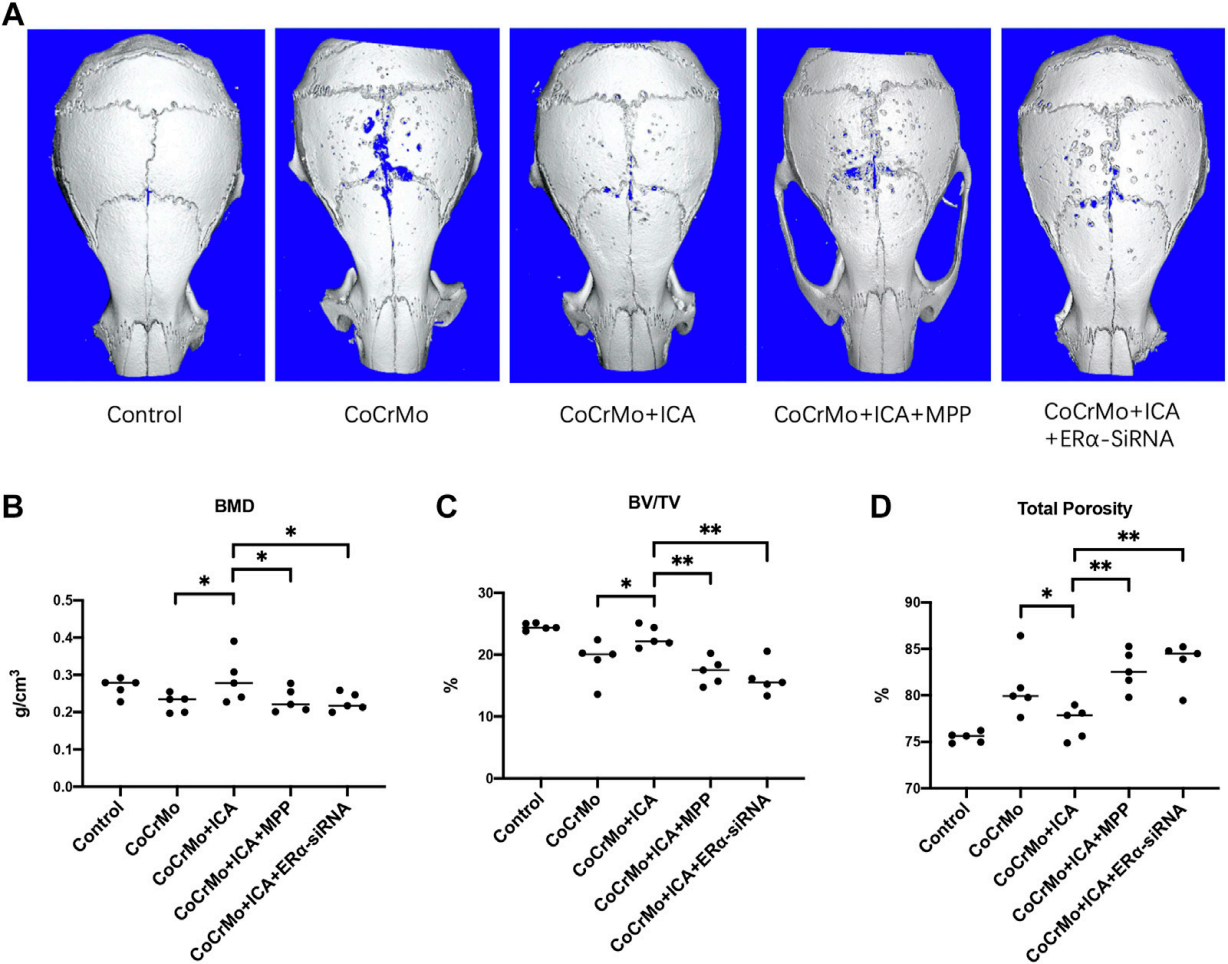
The protective effect of ICA on CoCrMo particle-induced mouse calvarial osteolysis was regulated by ERα. **(A)** Representative 3D reconstruction images for each group. No significant differences in BMD **(B**), BV/TV **(C)** or total porosity **(D)** were found between the ICA and control groups. The protective effect of ICA on BMD, BV/TV and total porosity were reversed by ERα antagonist and by ERα-siRNA interference **(B–D)**. * indicates *p* < 0.05, ** indicates *p* < 0.01, and *** indicates *p* < 0.001. There were five C57BL/6J mice in each group.

3D reconstruction of micro-CT images showed that CoCrMo particles induced profound changes in calvarial bone microarchitecture, as well as significantly decreased BMD (0.044 ± 0.034 g/cm^3^, *p* < 0.05) and BV/TV (−5.4 ± 2.5%, *p* < 0.01) (Figure 5). Consistent with the results from *in vitro* experiments, 3D images (Figure 5A) showed that ICA exerted a marked protective effect on CoCrMo particle-induced osteolysis. No significant differences in bone microstructure parameters were found between the ICA and control groups (Figures 5B–D). It was also noted that the protective effect of ICA on BMD, BV/TV and total porosity was reversed by ERα antagonist and by ERα-siRNA interference.

**FIGURE 6:**
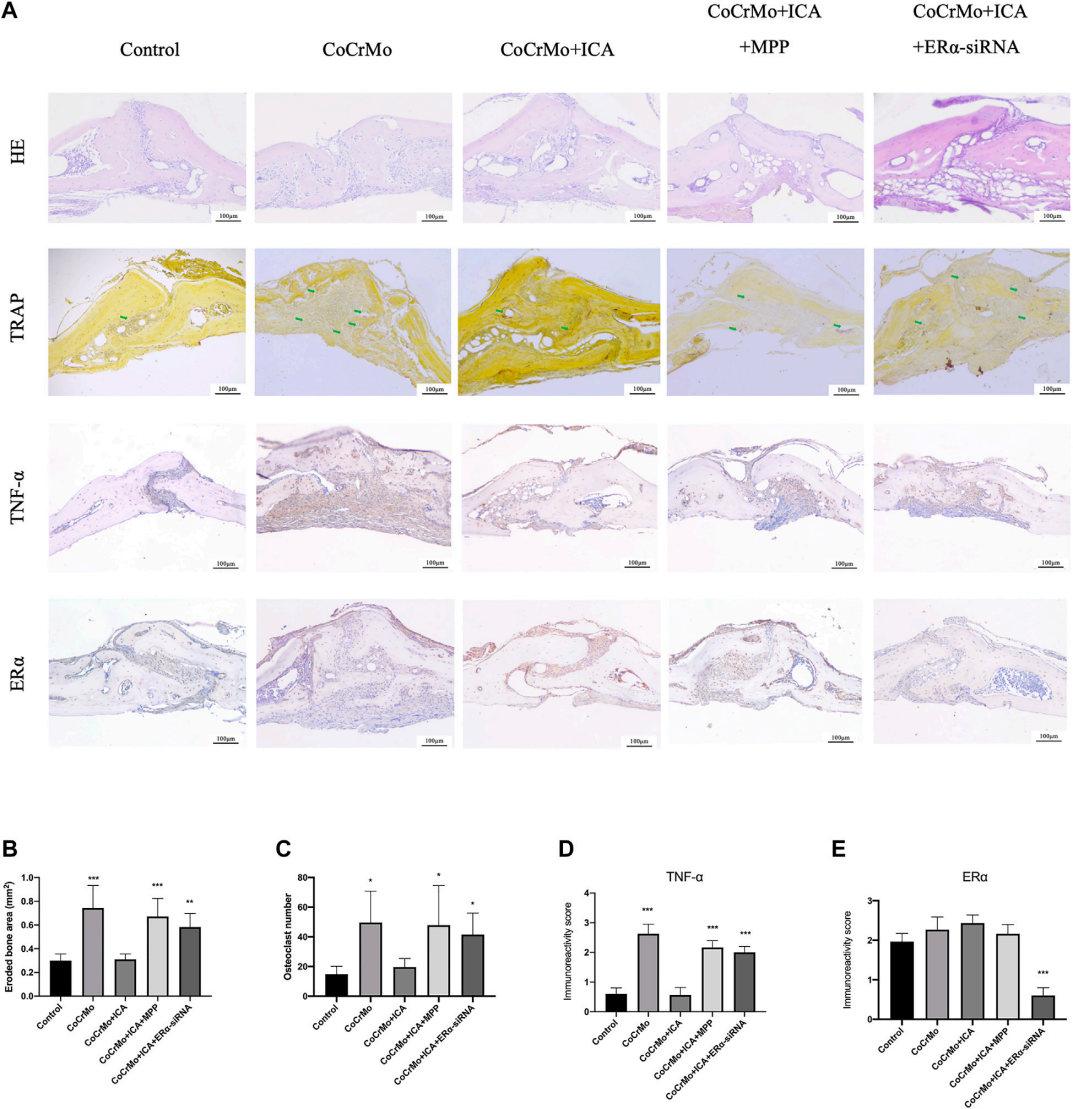
The protective effect of ICA on CoCrMo particle-induced osteolysis and local inflammatory infiltration in mouse calvaria was regulated by ERα. **(A)** Representative histological slide images of HE, TRAP, TNF-α, and ERα staining for each group at 10x magnification. The green arrows indicate osteoclasts. Quantitative measurements were obtained by calculating the eroded bone area **(B)**, TRAP-positive cell number **(C)**, TNF-α staining intensity **(D)**, and ERα staining intensity **(E)**. * indicates *p* < 0.05, ** indicates *p* < 0.01, and *** indicates *p* < 0.001. There were five C57BL/6J mice in each group.

HE and TRAP staining showed that ICA treatment suppressed CoCrMo wear particle-induced mouse calvarial osteolysis and osteoclastogenesis (Figure 6A). This was confirmed by quantitative measures of eroded bone area (−0.43 ± 0.19 mm^2^, *p* < 0.001) and of TRAP-positive cell number (−30.0 ± 19.4, *p* < 0.05) (Figures 6B,C). Significant increases in the eroded bone area and TRAP-positive cell numbers were found in the CoCrMo + ICA + MPP and CoCrMo + ICA + ERα-siRNA groups compared to the CoCrMo + ICA group. Representative images at a higher magnification of TRAP staining sections were provided in Supplementary Figure S2. Similar findings were observed for local inflammatory infiltration, as evaluated by TNF-α staining (Figure 6A) and subsequent semiquantitative analysis (Figure 6D). The efficiency of ERα knockout in the mice calvaria model by the injection of shRNA-ERα lentivirus was supported by the results of immunohistochemical staining for ERα in the calvarial sections (Figure 6A). Semiquantitative analysis showed that there was a significant decrease of the ERα immunoreactivity score in the ERα-siRNA targeting group when compared to the control group (Figure 6E).

## Discussion

It has been well documented that wear particles (including alumina ceramic, polyethylene, and alloy particles) play an important role in the development of postoperative periprosthetic osteolysis (Harris, 1994). To better mimic the clinical scenario, in this study LPS-free wear particles were made from a removed CoCrMo femoral head implant by using fabricated high-vacuum three-electrode direct current (Deng et al., 2021). Physical characteristics of the wear particles, including their chemical composition and diameter, are closely related to the severity of wear particle-induced macrophage inflammation response and osteolysis, with nanoscale alloy particles (<200 nm) having the biggest impact (Ding et al., 2012). The CoCrMo particles (150.2 ± 37.4 nm) used in the present study resulted in high levels of macrophage inflammation and osteolysis.

Although several cell types including macrophages, osteoblasts, osteoclasts and dendritic cells are involved in the development of wear particle-induced periprosthetic osteolysis, the activation of macrophages is widely considered to be the initial and vital step (Goodman et al., 2020). After being phagocytized, wear particles induce the secretion of proinflammatory cytokines (TNF-α and IL-6, etc.) by the macrophages, followed by macrophage M1 polarization and osteoclastogenesis. This eventually leads to a profound periprosthetic osteolysis (Goodman and Gallo, 2019). The molecular mechanism by which the wear particles are recognized by the macrophages and subsequently trigger the inflammation response is still not entirely clear. However, considerable evidence now suggests that NF-κB signaling is one of the pivotal pathways responsible for the wear particle-induced inflammatory activation and macrophage M1 polarization (Lin et al., 2017; Gao et al., 2018; Goodman et al., 2020). Furthermore, the NF-κB signaling pathway was proposed as the main target of potential medical therapies for periprosthetic osteolysis prevention and treatment (Hu et al., 2020). Consistent with this, significantly increased expression levels of TNF-α and IL-6 were observed in stained sections of clinical samples and in the *in vitro* experiments in the present study, along with marked activation of the NF-κB signaling pathway.

ICA shows well-known estrogen-like protective effects against RANKL- and estrogen deficiency-induced osteolysis, both *in vitro* and *in vivo* (Zhang et al., 2016; Ho et al., 2018; Xu et al., 2020). However, the influence of ICA on wear particle-induced periprosthetic osteolysis has only rarely been studied. Shao and others found that gavage-fed ICA protected against wear particle-induced osteolysis and also reduced the expression of TNF-α, IL-1β and IL-6 in a mouse calvarial model (Shao et al., 2015). Using micro-CT scanning, histological analysis and immunohistochemical analysis, the present study made similar findings to those of Shao et al. Subcutaneously implanted minipumps were also used here to obtain better accuracy and continuity of drug delivery, as well as to avoid the animal discomfort caused by gavage. The suppressive effect of ICA on the wear particle-induced upregulation of expression and secretion of TNF-α and IL-6 was further confirmed here in the experiments with BMDMs.

The mechanism that underlies the protective effect of ICA on CoCrMo particle-induced mouse calvarial osteolysis remains largely unknown. Multiple studies suggest the NF-κB signaling pathway plays an important role, with ICA exerting osteogenic and anti-osteoclastic effects not only on macrophages (Kim et al., 2018) but also on osteoblasts (Zhang et al., 2017) and osteoclasts (Xu et al., 2019). Furthermore, bioinformatics analysis revealed that RELA, NFKBIA, and IKBKB, which all belong to the NF-κB family, were hub genes shared between ICA-targeted genes and osteoporosis (Yu et al., 2020). Consistent with this, we found the inhibitory effect of ICA on the CoCrMo wear particle-induced inflammatory response of BMDMs was also mediated by NF-κB signaling. Additionally, it was reported that wear particle-induced M1 macrophages contribute to enhanced osteoclast formation and hence the regulation of macrophage polarization was suggested as a potential strategy to suppress wear particle-induced osteolysis (Zhu et al., 2019). In the present study, the percentage of M1 polarization amongst BMDMs decreased significantly following ICA treatment compared to the CoCrMo control group. Taken together, we propose that ICA suppresses CoCrMo wear particle-induced osteolysis and local inflammatory responses *via* downregulation of the NF-κB signaling pathway.

ICA is a phytoestrogen and hence its protective effect on bone tissue is thought to be mediated mostly through ERα. A recent study found marked activation of ERα and elevated osteogenic activity in both pre-osteoblastic cells and mature osteoblasts after ICA treatment (Zhou et al., 2021), indicating potential links between ERα and the bone preserving effect of ICA. Previous studies also reported that ICA stimulated osteoblast differentiation of bone marrow stromal cells *via* activation of the ERα signaling pathway, which could be reversed by ERα antagonist (Wei et al., 2016; Li et al., 2018). Additionally, bioinformatic analysis in the present study found that ERα has a close relationship with TNF-α and IL-6, indicating that ERα is a potential regulatory target for CoCrMo particle-induced macrophage activation. Thus, we hypothesize that the inhibitory effect of ICA on the CoCrMo particle-induced inflammatory activation of macrophages and on periprosthetic osteolysis is mediated by ERα. IF assay and flow cytometry analysis demonstrated that ICA induced a marked increase in protein expression of phospho-ERα and in translocation of ERα from the cytoplasm into the nucleus. These occurred concomitantly with the multiple inhibitory effects of ICA on wear particle-related activation of the NF-κB signaling pathway, secretion of proinflammatory factors, and macrophage polarization. The ERα-specific antagonist MPP and also ERα-siRNA interference were used in the present study to further test this hypothesis. The protective effects of ICA against the CoCrMo particle-induced inflammatory response in macrophages and on osteolysis were significantly reversed by both of these interventions in our *in vivo* and *in vitro* experiments. Therefore, we propose that ICA reduces wear particle-induced activation of inflammation and osteolysis by inhibiting the ERα-mediated NF-κB signaling pathway in macrophages.

To the best of our knowledge, this is the first study to investigate the relationship between the protective effect of ICA on wear particle-induced osteolysis and modulation of the ERα pathway. Moreover, only one previous animal study has investigated the relationship between wear particle-induced activation of macrophages and ERα(Nich et al., 2013). Although the authors proposed that ERα may be considered as a future therapeutic target for particle-induced osteolysis, they found that pre-treatment with the non-specific ER antagonist ICI 182780 resulted in consistent down-regulation of particle-induced TNF-α mRNA expression in macrophages. ICI 182780 is known to block both ERα and ERβ, although ERβ shows opposite effects to ERα and supports pro-inflammatory immunity (Koenig et al., 2017). Thus, we propose that inhibition of ERβ by this non-specific ER antagonist may explain the contradictory results between the previous study and ours. However, more evidence is needed to clarify this issue.

The present study was subject to certain limitations. Firstly, the specific knock-down of macrophage ERα in the mice calvaria *via* ERα-siRNA interference could not be fully verified in the present study. This established method of our group has been used in several earlier studies (Zhang et al., 2018; Qiu et al., 2020), and marked inhibition of ERα expression in mice calvarial sections was found after ERα-siRNA lentivirus injection. However, unspecific knock-down effect in other cells from the surrounding tissue addition to the macrophages has potential influence on the decreased therapeutic effect of ICA, as it was reported that ICA promoted osteogenic differentiation of bone marrow stromal cells by activating the ERα(Wei et al., 2016). Thus, more direct evidence of the knockdown of ERα in host macrophages of C57/BL6J mice is strongly needed. The application of the gene-editing mice with specific ERα knockdown in macrophages in future studies could provide solid evidence *in vivo* to fully support our hypothesis. Secondly, although previous studies showed that ICA activated both classical (genomic) and extra-nuclear (non-genomic) ERα signaling cascades (Arnal et al., 2017; Ho et al., 2018), these were not fully assessed in the present study. Further ongoing research by our group is aimed at gaining a better understanding of the relationship between the protective effect of ICA on wear particle-induced osteolysis and the ERα non-genomic pathway. Furthermore, we didn’t investigate the influence of ICA intervention on macrophage polarization *in vivo* in the present study. We plan to perform the flow cytometry analyses for the soft tissue covering the mice’s parietal bones in future studies to further investigate this issue (Eger et al., 2018). Lastly, although the wear particles-induced mice calvarial osteolysis model is currently the most widely used animal model which aimed to mimic the clinical scenario of wear particle-induced periprosthetic osteolysis (Goodman et al., 2020), the osteolysis process lasted for only 2 weeks, representing more likely an acute inflammatory process rather than a chronic one. Thus, the clinical relevance of the murine calvaria model should be considered with caution. Besides, although our *in vitro* studies protocols are similar to previous studies (Qiu et al., 2020; Hu et al., 2021) concerning the wear particles-induced inflammation and osteolysis, we must admit that future cell studies with a longer stimulation time are needed to further investigate the protective effect of ICA on chronic inflammatory reactions caused by wear particles.

## Conclusion

In conclusion, ICA suppresses wear particle-induced activation of inflammation and osteolysis via ERα-mediated down-regulation of the NF-κB signaling pathway in macrophages. ICA therefore has potential application as a non-hormonal therapy for wear particle-induced periprosthetic osteolysis, without inducing undesirable estrogen-dependent transcriptional events.

## Data Availability

The original contributions presented in the study are included in the article/Supplementary Material, further inquiries can be directed to the corresponding authors.

## Abbreviations

BMDMs, bone marrow-derived macrophages; ELISA, enzyme-linked immunosorbent assay; H&E, hematoxylin and eosin; ICA, icariin; IF, immunofluorescence; MPP, methyl-piperidino-pyrazole; PPI, protein-protein interaction; ROI, region of interest; TRAP, tartrate-resistant acid phosphatase.
