# Supplementary figures and images for: Icariin Alleviates Wear Particle-Induced Periprosthetic Osteolysis via Down-Regulation of the Estrogen Receptor α-mediated NF-κB Signaling Pathway in Macrophages
Source: Front Pharmacol. 2021 Nov 3;12:746391. doi: 10.3389/fphar.2021.746391 (PMC8595199; doi:10.3389/fphar.2021.746391)

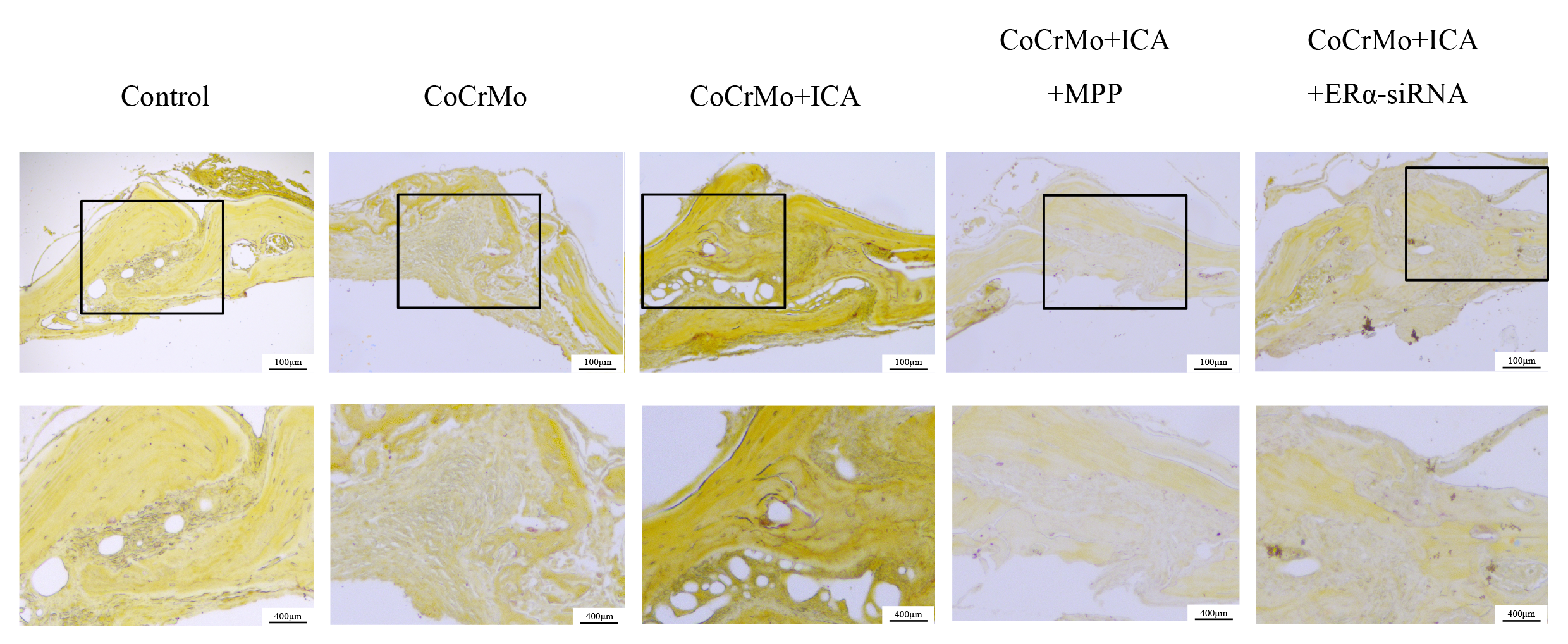

Supplement: Supplementary file 1 [file Image2.TIF]

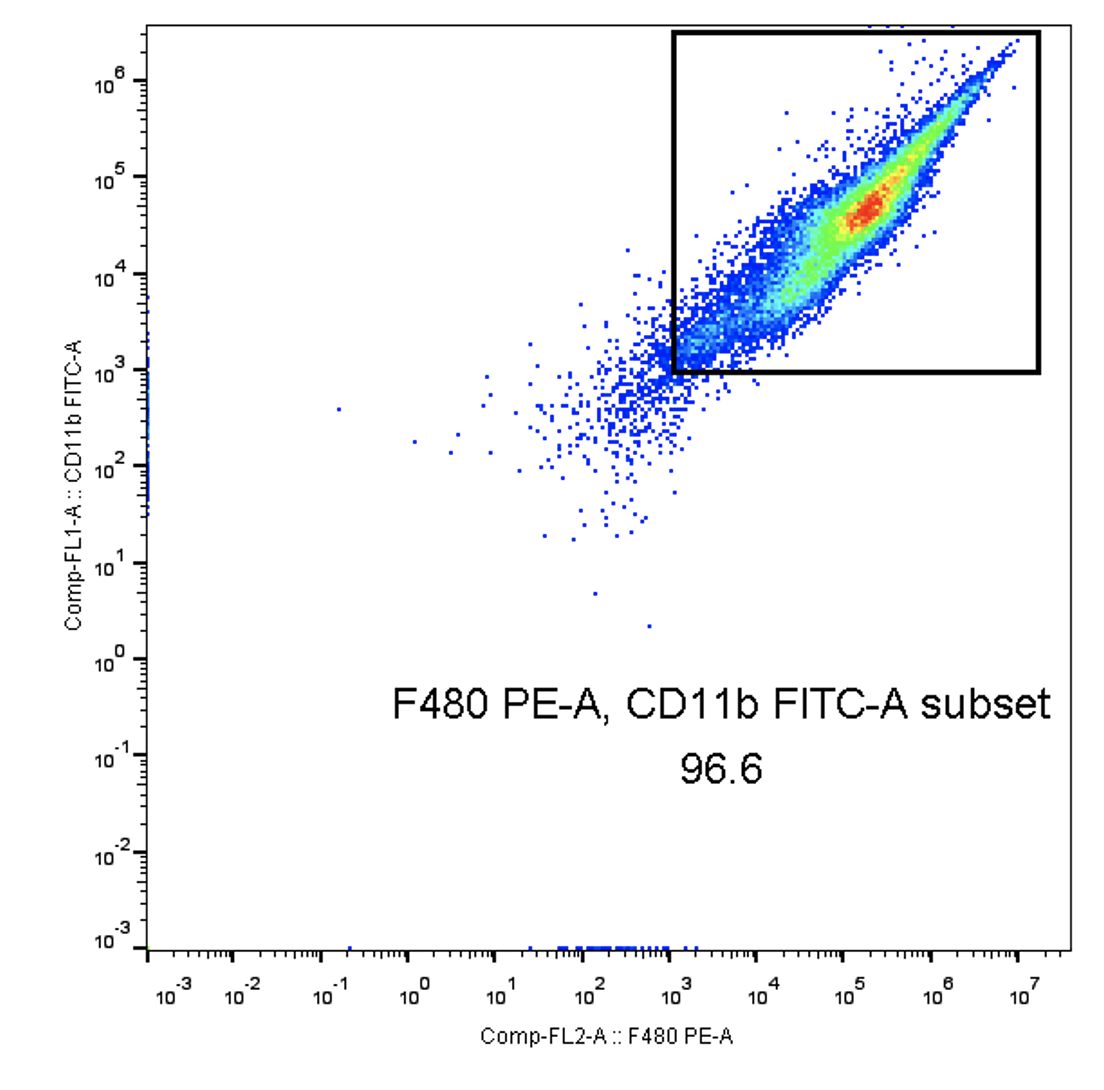

Supplement: Supplementary file 2 [file Image1.TIF]
