# Supplementary material for: Icariin Alleviates Wear Particle-Induced Periprosthetic Osteolysis via Down-Regulation of the Estrogen Receptor α-mediated NF-κB Signaling Pathway in Macrophages
Source: Front Pharmacol. 2021 Nov 3;12:746391. doi: 10.3389/fphar.2021.746391 (PMC8595199; doi:10.3389/fphar.2021.746391)
Supplement: Supplementary file 3 [file DataSheet2.docx]

**Supplementary Figure legends**

Supplementary Fig 1. Representative flow cytometry results (F4/80+ CD11b+) of BMDMs that were isolated from the bone marrow of mice femurs.

Supplementary Fig 2. Representative images at a higher magnification of TRAP staining in Fig 6A.
