# Supplementary material for: Icariin Alleviates Wear Particle-Induced Periprosthetic Osteolysis via Down-Regulation of the Estrogen Receptor α-mediated NF-κB Signaling Pathway in Macrophages
Source: Front Pharmacol. 2021 Nov 3;12:746391. doi: 10.3389/fphar.2021.746391 (PMC8595199; doi:10.3389/fphar.2021.746391)
Supplement: Supplementary file 4 [file DataSheet1.docx]

Table S1. Demographic data

| Patients | Diagnosis | Age (years) | Sex | Time after the primary THA (years) |
| --- | --- | --- | --- | --- |
| 1 | Implant aseptic loosening | 87 | Male | 17 |
| 2 | Implant aseptic loosening | 78 | Male | 8 |
| 3 | Implant aseptic loosening | 82 | Female | 21 |
| 4 | Implant aseptic loosening | 83 | Female | 13 |
| 5 | Femoral neck fracture | 85 | Male | N.A. |
| 6 | Femoral neck fracture | 89 | Male | N.A. |
| 7 | Femoral neck fracture | 76 | Female | N.A. |
| 8 | Femoral neck fracture | 69 | Female | N.A. |

Table S2. Primers used in the present study

| TNF-α | Forward | CCTGTAGCCCACGTCGTAG |
| --- | --- | --- |
|  | Reverse | GGGAGTAGACAAGGTACAACCC |
| IL-6 | Forward | CTGCAAGAGACTTCCATCCAG |
|  | Reverse | AGTGGTATAGACAGGTCTGTTGG |
| GAPDH | Forward | TGTGTCCGTCGTGGATCTGA |
|  | Reverse | TTGCTGTTGAAGTCGCAGGAG |

Table S3. Details of the antibodies used for WB, immunohistochemistry and immunofluorescence staining

| Antibodies | Brands |
| --- | --- |
| p-ERα Ser118 | rabbit, ab32396, abcam |
| p-ERα Ser167 | rabbit, ab131105, abcam |
| ERα | rabbit, ab32063, abcam |
| p-IKKβ | rabbit, 2697, Cell Signaling Technology |
| IKKβ | rabbit, 8943, Cell Signaling Technology |
| p-p65 | rabbit, 3033, Cell Signaling Technology |
| p65 | rabbit, 8242, Cell Signaling Technology |
| p-IκBα | rabbit, 2859, Cell Signaling Technology |
| IκBα | mouse, 4814, Cell Signaling Technology |
| GAPDH | rabbit, 5174, Cell Signaling Technology |
